# Fibromatosis Colli: A Case Report

**DOI:** 10.7759/cureus.47308

**Published:** 2023-10-19

**Authors:** Nouf F Aljahdali, Ahmed A Alolah, Amer A Alghamdi, Falwah F Alharthi, Narjis J Aljaziri

**Affiliations:** 1 Pediatrics Emergency Medicine, King Abdullah Specialized Children’s Hospital (KASCH), Riyadh, SAU; 2 Emergency Medicine, King Abdulaziz Medical City, Riyadh, SAU; 3 Medicine, King Saud Bin Abdulaziz University for Health Sciences, Riyadh, SAU

**Keywords:** torticollis, pseudotumor, sternocleidomastoid tumor, ultrasound, fibromatosis colli

## Abstract

Fibromatosis colli is an infrequent and self-limiting disorder in newborns with an unknown etiology. It usually presents with an abnormal head position or cervical swelling. The clinical diagnosis of fibromatosis colli is important to avoid unnecessary invasive interventions. The condition is treated conservatively with physiotherapy. In this article, we report the case of a two-month-old infant who presented with an abnormal head position and was diagnosed with fibromatosis colli based on ultrasonographic examination, which is the non-invasive diagnostic intervention of choice.

## Introduction

Fibromatosis colli, or congenital torticollis, is an infrequent and self-limiting disorder caused by a nonmalignant tumor in infants' sternocleidomastoid muscles (SCMs). It is a rare condition, occurring in around 0.4% of live births and mainly in males. Most cases affect the right side of the neck [1]. Despite its unknown etiology, it is generally believed that the pseudotumor results from an underlying muscle injury that can occur in utero due to poor fetal head positioning [2]. The tumor can also develop from birth-related trauma. The diagnosis is mainly clinical and is often confirmed by ultrasound [1]. The condition is primarily managed through physiotherapy.

## Case presentation

We present a two-month-old female brought by her mother to the emergency department to evaluate an abnormal head position. The mother has noticed that the child cries when she tries to move her head. The head of the newborn always tilts to the left. There seemed to be discomfort and limited mobility in the neck, resulting in pain. The mother has noticed these changes whenever she tried to change the baby's clothes for the last two weeks, albeit briefly. There was no history of fever, trauma, or abnormal movements. The mother did not give the baby any medication. There was no history of neuromuscular or congenital disorders in the family. Baby is a product of non-instrumental, normal vaginal delivery; she is a single gestation. Her birth weight was 3.1 kg. The baby looked well and was active without dysmorphic features. The head is inclined to the left side without visible swelling or skin changes. On palpation of the neck, there is no muscle hardening, masses, or enlarged lymph nodes. However, passive movement of the neck made her irritable. Bilateral upper and lower arms moved freely with positive moral reflexes. Other systematic examinations were unremarkable.

**Figure 1:**
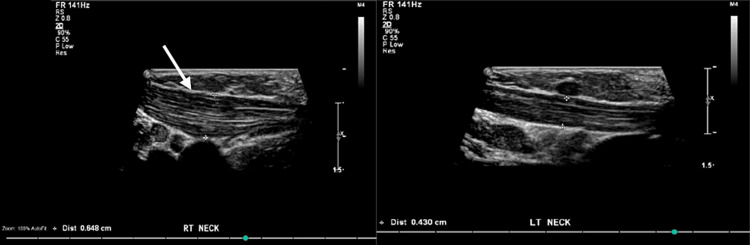
Ultrasound appearance of homogeneous fusiform hypertrophy of the right SCM muscle (white arrow) compared to the left SCM muscle.

The differential diagnosis included SMC hematoma, tumor, lymphadenopathy, and fibromatosis colli. The clinical presentation of the infant, including the report by the mother concerning pain and discomfort when she tries moving her head, which leans to the left, raised suspicion for fibromatosis colli. The diagnosis was confirmed via a neck ultrasound, which revealed that the right SCM was thicker than the left, measuring 0.6 cm versus 0.4 cm on the left side (Figure 1). Further, a head ultrasound was conducted to rule out bleeding or abnormal pathology. The otolaryngologist did a nasal scope and showed no posterior pharyngeal wall bulge. The vocal cords are mobile and symmetrical, with no signs of obstructions or abscesses in the airways or neck spaces. The results of the lab tests conducted did not yield any significant findings. The patient was admitted to the general pediatrics department for conservative therapy and physiotherapy for fibromatosis colli.

## Discussion

Fibromatosis colli was first described as the sternocleidomastoid tumor of childhood (SCMT) [3]. It is categorized as a benign fibroblastic proliferation of the sternocleidomastoid muscle [4]. It is a rare condition that affects around 0.4% of live births [5]. The WHO classification of soft tissue tumors classifies fibromatosis colli as a nonmalignant fibroblastic tumor. These tumors often occur in infants between two to four weeks; however, some cases have been reported where the tumors present in older infants and young adults [2,6]. The infants look normal at birth and develop a unilateral neck mass two to four weeks after birth. Normally, 73% of the cases occur on the right side, and they mostly affect infants of the male gender [7]. Fibromatosis colli is often associated with hard labor, the use of breech delivery, forceps, or birth trauma. The causes of fibromatosis colli are likely linked to a decrease in blood flow during pregnancy or fetal malposition in utero [1]. Other causes of this condition include heredity and infection. The infection hypothesis can be explained by considering the reduction of blood flow to the SCM due to developing a septic thrombus.

Fibromatosis colli presents mainly during the newborn period with cervical swelling and abnormal head position [8]. Physical examination revealed a non-tender mass on the right side more than the left and rarely bilateral [8]. Its differential diagnosis includes congenital lesions, such as branchial cysts and thyroglossal cysts; inflammatory lesions such as tuberculous lymphadenitis; and neoplastic conditions that could be nonmalignant (hemangioma, cystic hygroma) or malignant (neuroblastoma, rhabdomyosarcoma, and lymphoma). Fibromatosis colli was diagnosed via US and showed a fusiform or oval subcutaneous formation developed at the expense of the upper or middle third of the sternocleidomastoid [8] and, recently, the role of FNA cytology, which revealed singly scattered and loose clusters of nonmalignant fibroblasts with a moderate amount of unipolar to bipolar cytoplasm and plumped, ovoid nuclei [9]. The condition is treated conservatively with physiotherapy, and, in the meantime, there is a general improvement in signs and symptoms after two to three months of therapy [10].

Regarding detection, if ultrasound results are inconclusive, further evaluation of the pseudotumor can be conducted using CT scans. However, it is important to note that CT scans expose the newborn to ionizing radiation. The CT scan typically reveals an enlargement of the SCM with normal surrounding structures, showing isoattenuation [11]. On the other hand, MRI imaging provides valuable insights. It displays a uniform and hyperintense pseudotumor within the SCM T2-weighted sequences, in contrast to the appearance of normal muscle tissue. Additionally, MRI helps rule out any vascular or airway compression, thus negating other possible conditions [12].

While performing a biopsy on the mass is not recommended, cytology examinations show fibroblasts with mild features, along with degenerated and atrophic smooth muscle. There is no indication of bleeding or inflammation. The background exhibits muscle giant cells, collagen, and bland, bare nuclei [12]. Treatment primarily leans toward a conservative approach. Most cases have been managed conservatively, involving observation and physiotherapy. This approach entails gently turning the newborn's neck toward the side of the lesion around five to six times a day, maintaining that position for a brief duration [13]. This practice is maintained over several months until the swelling starts to diminish. Fibromatosis colli often resolves spontaneously within four to eight months, even without treatment. However, in refractory cases (less than 10% of occurrences), alternative treatments such as botulinum toxin type A or surgical tenomyotomy could be pursued [12].

The treatment and management of this condition can be achieved through conservative management, medical intervention, or surgical intervention. Conservative management is done through observation and physiotherapy. Since some minor condition cases resolve over time, a watchful waiting approach is recommended, especially for cases where the condition does not cause significant functional limitations or discomfort [1]. Regarding physiotherapy, the newborn's neck can be turned gently and repeatedly to help improve neck mobility and resolve the pseudotumor. A study revealed that nearly 95% of infants who had minimal limitations experienced enhanced mobility after undergoing a four-week active home stimulation program [14]. Moreover, approximately 91% of infants with more significant limitations reported positive outcomes following three targeted physiotherapy sessions conducted over a period of three to four months [15]. When conservative measures prove ineffective, medical interventions can be adopted. Botulinum toxin type A injections can help the muscles relax and shrink the pseudotumor [1]. Surgical intervention, specifically tenotomy, was suggested for infants who did not observe any enhancements in mobility after undergoing therapy for one year or for those who sought treatment after reaching 12 months of age [15].

## Conclusions

Fibromatosis colli, or congenital torticollis, is an infrequent and self-limiting disorder characterized by a nonmalignant tumor in infants' SCMs. Although its etiology remains unknown, it is believed to stem from injuries sustained either during fetal development in utero or due to birth-related trauma. Although the condition is relatively uncommon (0.4% of live births), most cases occur in males. Clinically, the condition's presentations include abnormal head position, limited neck mobility, and discomfort. Diagnostic evaluation entails clinical assessments conducted through ultrasound, while treatment and management can be through conservative management, medical intervention, or surgical intervention.
